# Early Postpartum Screening: Predictive Value of Edinburgh Postnatal Depression Scale (EPDS) Scores on Day 3 for Depression at One Month

**DOI:** 10.7759/cureus.94716

**Published:** 2025-10-16

**Authors:** Mizuki Ono, Yasushi Kurihara, Kohei Kitada, Mie Tahara, Akihiro Hamuro, Akemi Nakano, Takuya Misugi, Daisuke Tachibana

**Affiliations:** 1 Obstetrics and Gynecology, Osaka Metropolitan University Graduate School of Medicine, Osaka, JPN

**Keywords:** depression, early screening, edinburgh postnatal depression scale, post partum, pregnancy

## Abstract

Objective

Postpartum depression often develops within the first few months after delivery, with many cases emerging within four weeks. Although the Edinburgh Postnatal Depression Scale (EPDS) is commonly administered at one month postpartum in Japan, earlier onset highlights the need for earlier screening. This study aimed to examine the relationship between EPDS scores at postpartum day 3 and at one month, and to evaluate the predictive value of early screening.

Methods

We retrospectively analyzed 559 women who delivered at our hospital between January and December 2021 and attended their one-month postpartum checkup. EPDS was administered to all participants on postpartum day 3 and at one month postpartum. A cutoff of ≥9 points was used to define positivity. Correlation was assessed using Spearman’s test, and receiver operating characteristic (ROC) analysis was performed to determine the optimal cutoff score on day 3 for predicting positivity at one month.

Results

At one month postpartum, 41 women (7.3%) were EPDS-positive. On postpartum day 3, 131 women (23.4%) were positive, of whom 22 (16.8%) remained positive at one month. Among 428 women negative at day 3, 19 (4.4%) converted to positive at one month. EPDS scores on day 3 were significantly correlated with those at one month (r = 0.476, p < 0.001). ROC analysis identified 5 points as the optimal cutoff on day 3 for predicting positivity at one month.

Conclusion

EPDS scores at postpartum day 3 are significantly correlated with those at one month and may help identify women at risk for postpartum depression earlier than the conventional one-month screening. Even women scoring 5-8 points at day 3, though below the traditional cutoff, showed an increased risk of later conversion and warrant careful follow-up and supportive interventions.

## Introduction

Postpartum depression typically develops within the first few months after delivery, with the first four weeks recognized as the peak onset period [1]. Approximately 15% of women during pregnancy and 14% after childbirth are reported to experience perinatal depression [2]. The proportion of suicide among maternal deaths has also been increasing, posing a serious threat to maternal health [3]. Moreover, maternal mental health has been linked not only to child growth and development but also to the risk of child abuse [4,5]. Thus, maintaining maternal psychological well-being in the postpartum period is of critical importance for both mother and child.

The Edinburgh Postnatal Depression Scale (EPDS) is widely used to assess maternal mental health during the puerperium [6]. A score of 9 or higher is generally considered positive, warranting psychiatric referral or consideration of psychotropic medication when appropriate. However, many mothers are reluctant to seek psychiatric care or to take medication while breastfeeding [7]. The consensus guidelines of the Japan Society of Perinatal Mental Health recommend EPDS screening at one month postpartum, yet many cases manifest earlier, underscoring the need for earlier intervention.

At our institution, the EPDS is administered on postpartum day 3 to facilitate early detection and timely intervention, followed by outpatient visits or collaboration with public health nurses, depending on the results. The present study aimed to clarify the relationship between EPDS scores at postpartum day 3 and at one month postpartum, and to evaluate the utility of early screening.

## Materials and methods

We included women who delivered at Osaka Metropolitan University Hospital between January and December 2021 and attended their one-month postpartum checkup. Eligible participants were women who delivered at our hospital at ≥22 weeks of gestation. The EPDS was administered to all participants on postpartum day 3 and at one month postpartum. At two weeks postpartum, the EPDS was administered only to those requiring medical follow-up, those with psychological instability, or those requesting assessment due to childcare concerns. Based on our institutional policy, an EPDS score of ≥9 was defined as positive and used as the threshold for medical intervention. Participants were classified into positive (≥9 points) and negative (≤8 points) groups, and the proportion of positive EPDS results at one month was calculated. Women who did not attend the one-month postpartum checkup were excluded.

Participants with severe psychiatric symptoms were rare, and most of them had EPDS scores ≥9 on postpartum day 3. As these cases represent the real-world perinatal population encountered in clinical practice, they were intentionally included in the analysis rather than excluded. The correlation between EPDS scores at postpartum day 3 and at one month was analyzed using Spearman’s correlation test. In addition, we determined the cutoff value for women with a negative EPDS on day 3 who later converted to positive at one month. To evaluate the predictive ability of EPDS scores on postpartum day 3 for positive results at one month, receiver operating characteristic (ROC) curve analysis was performed. The area under the curve (AUC) and 95% confidence intervals (CIs) were calculated. Statistical analyses were performed using IBM SPSS Statistics for Windows, Version 21.0 (IBM Corp., Armonk, New York, United States), with a significance level of 0.05.

Ethical considerations

This study was conducted in accordance with the Declaration of Helsinki and the Ethical Guidelines for Medical and Health Research Involving Human Subjects. The study protocol was approved by the Ethics Committee of Osaka Metropolitan University Hospital (approval no. 2024-150).

## Results

**Figure 1:**
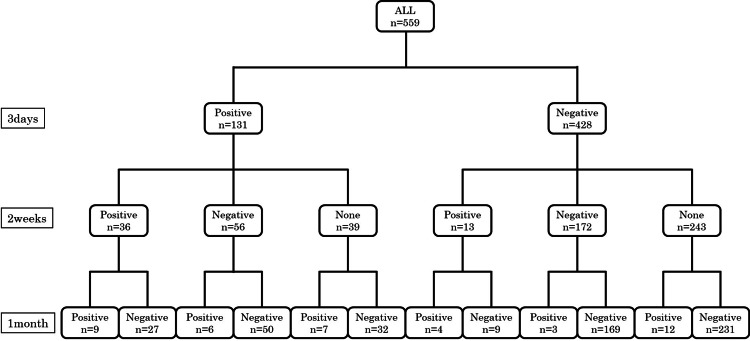
Flowchart of EPDS assessments at 3 days, 2 weeks, and 1 month postpartum. All 559 participants underwent the Edinburgh Postnatal Depression Scale (EPDS) assessment at 3 days, 2 weeks, and 1 month postpartum. The numbers of participants classified as positive, negative, or not assessed (none) at each time point are shown.

A total of 559 women were analyzed. At one month postpartum, 41 (7.3%) were EPDS-positive and 518 (92.7%) were negative. On postpartum day 3, 131 (23.4%) were positive, of whom 22 (16.8%) remained positive at one month. Among the 428 women who were negative on day 3, 19 (4.4%) converted to positive at one month (Figure 1).

**Figure 2:**
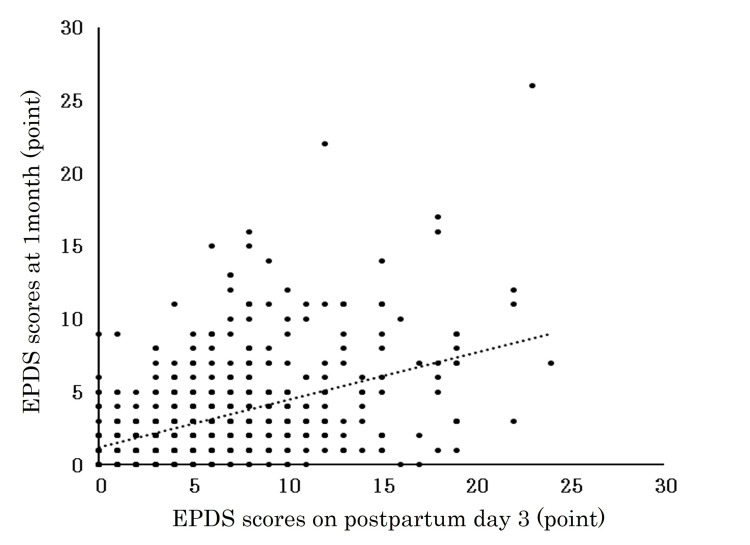
Conversion rate to EPDS positivity at 1 month postpartum by early postpartum EPDS scores. Each point represents an individual case. The dashed line indicates the line of equality. A positive correlation suggests that higher Edinburgh Postnatal Depression Scale (EPDS) scores in the early postpartum period tend to persist at 1 month. EPDS scores on postpartum day 3 showed a significant positive correlation with scores at one month (Spearman’s correlation test, r = 0.476, p < 0.001).

The proportion of positive cases at one month was significantly higher among those who were positive on day 3 (p < 0.001). EPDS scores on postpartum day 3 showed a significant positive correlation with scores at one month (r = 0.476, p < 0.001) (Figure 2).

**Figure 3:**
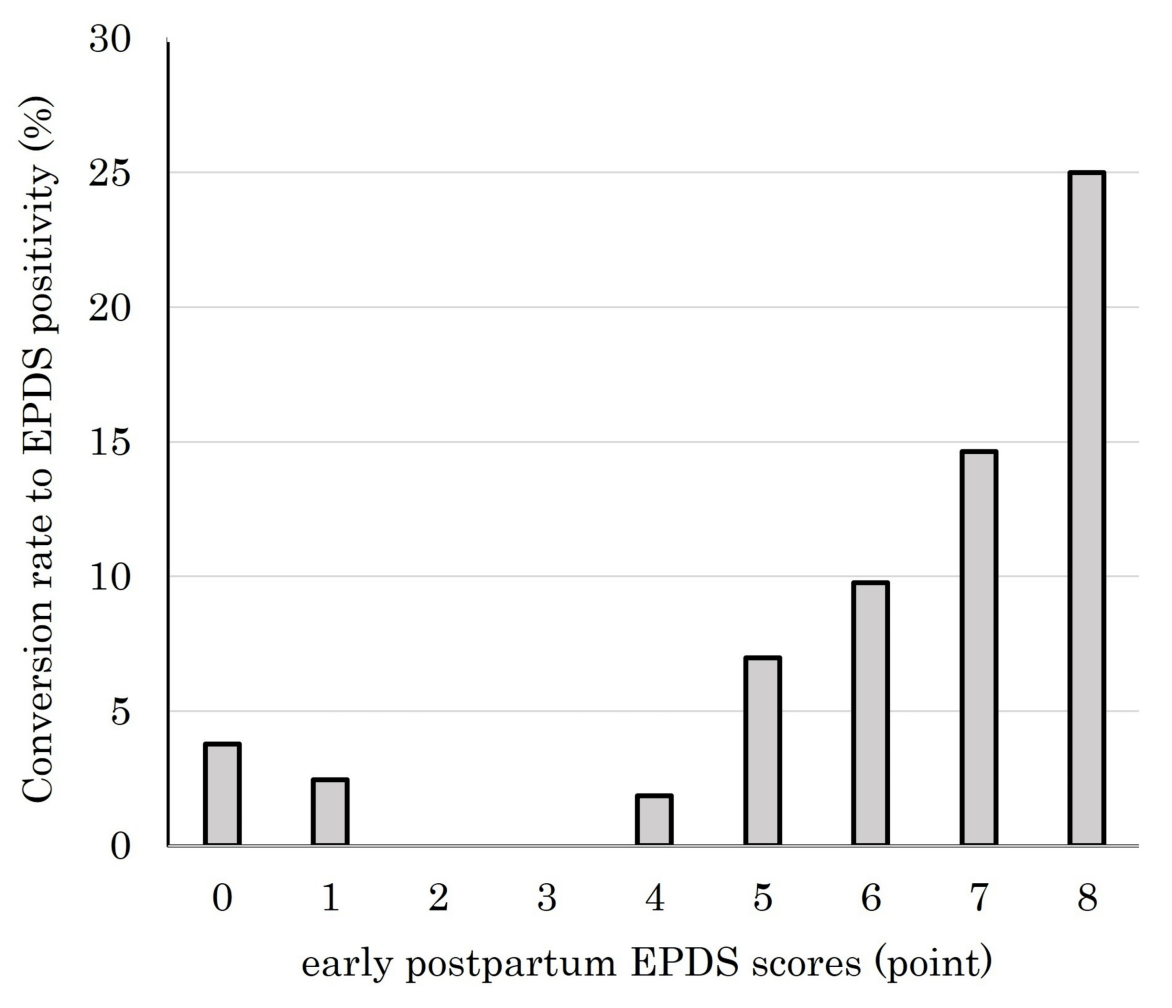
Conversion rate to EPDS positivity at 1 month postpartum by early postpartum EPDS scores. The bar graph shows the proportion of participants who became positive on the Edinburgh Postnatal Depression Scale (EPDS, score ≥9) at 1 month postpartum, stratified by their early postpartum EPDS scores (0–8 points).

**Figure 4:**
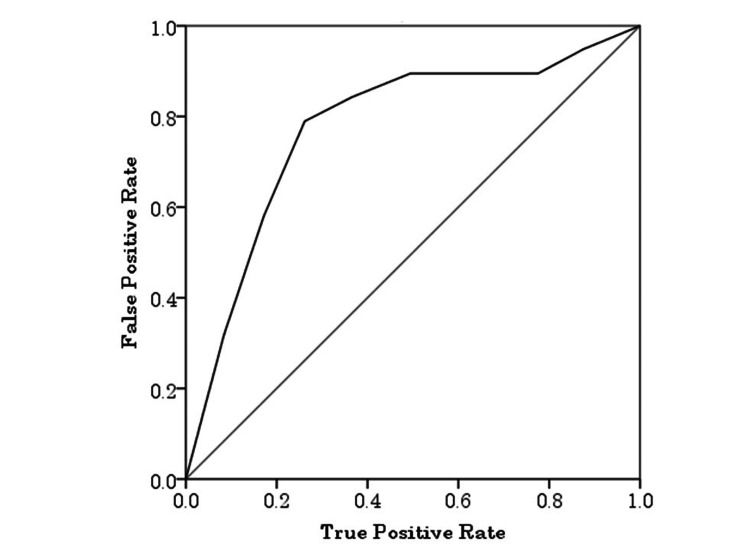
Receiver operating characteristic (ROC) curve for predicting EPDS positivity (score ≥9) at 1 month postpartum based on early postpartum EPDS scores. The area under the curve (AUC) was 0.754 (95% CI: 0.644-0.864), indicating good predictive ability. The optimal cutoff value was 5, yielding a sensitivity of 71.4% and specificity of 74.5%. EPDS: Edinburgh Postnatal Depression Scale

Furthermore, at 1 month postpartum, the conversion rate to EPDS positivity (score ≥9) increased in proportion to the early postpartum EPDS scores. As shown in Figure 3, women with higher EPDS scores within the first few days after delivery were more likely to become positive at 1 month compared with those with lower scores. ROC curve analysis revealed that EPDS scores on postpartum day 3 significantly predicted positive screening results at one month postpartum, with an area under the curve (AUC) of 0.754 (95% CI: 0.644-0.864, p < 0.001). Using a cutoff score of 5, the sensitivity was 71.4% and the specificity was 74.5% (Figure 4).

## Discussion

To our knowledge, this is the first study to describe sequential changes in EPDS scores during the postpartum period. EPDS scores at postpartum day 3 were significantly correlated with those at one month, suggesting that women at risk for postpartum depression can be identified early in the puerperium, thereby enabling earlier medical intervention. Importantly, these results may support earlier intervention and a re-evaluation of current one-month screening practices. In this study, women with high EPDS scores on postpartum day 3 were simply encouraged to attend their two-week checkup, and no active psychiatric or medical interventions were provided at that stage. Therefore, we believe that the influence of such encouragement on the results was minimal. Nevertheless, the present findings suggest that earlier or more proactive support may be considered in future practice for women identified as high-risk based on early EPDS screening.

Postpartum depression is characterized by persistent stress and low mood, often accompanied by fatigue, emotional instability, anergia, poor appetite, guilt, and social withdrawal [8]. Previous studies have identified several risk factors, including (i) endocrine factors such as abrupt changes or withdrawal of estrogen, (ii) perinatal complications such as preterm birth and gestational diabetes, (iii) unstable environments such as domestic violence or abuse, and (iv) educational and socioeconomic factors [9-13]. Given the multifactorial nature of maternal mental health, comprehensive evaluation and intervention are essential. Our hospital, as a tertiary perinatal center, manages many complicated cases in which maternal psychological burden due to perinatal complications is expected to be substantial. Nevertheless, in this study, only 44 women (7.7%) scored ≥9 on the EPDS at one month, which is about half the incidence generally reported (~15%) [2]. This may be attributable to our proactive support measures - such as frequent outpatient follow-ups and public health nurse visits - initiated after high EPDS scores at postpartum day 3, potentially preventing symptom progression or clinical manifestation.

Importantly, 19 women (4.4%) with negative EPDS scores (≤8) on postpartum day 3 converted to positive (≥9) at one month. This suggests that psychological distress and childcare burden may emerge after discharge, progressing to postpartum depression. Many of these women scored between 5 and 8 on day 3, indicating the presence of a “subthreshold” or “potential high-risk” group not captured by the conventional cutoff (≥9 or ≥10). Levis et al. reported in a large individual-participant data meta-analysis (58 studies, >15,000 participants) that an EPDS cutoff of ≥11 provided sensitivity of 81% and specificity of 88%, while a cutoff of ≥10 yielded sensitivity of 85% and specificity of 84%, making it more suitable for early screening [13]. Our findings support re-evaluating scores of 5-8 as a “gray zone” or “latent high-risk group.” Even when the day 3 score is below the conventional cutoff, women scoring ≥5 warrant continued monitoring and may benefit from non-invasive interventions (e.g., psychological support, traditional herbal medicine) from a preventive standpoint.

Breastfeeding is closely associated with both physical and psychological burden. Frequent feeding and sleep deprivation due to nighttime awakenings are known contributors to breastfeeding difficulties [14]. Conversely, oxytocin released during breastfeeding fosters maternal bonding and psychological stability. Mothers with depressive symptoms have been reported to show lower oxytocin levels during breastfeeding [15-17], suggesting a bidirectional relationship between mental health and breastfeeding. Frequent outpatient follow-up therefore provides an opportunity to alleviate breastfeeding difficulties, offer guidance, and support maternal psychological stability.

Limitations

This study has several limitations. First, it was conducted at a single tertiary perinatal center, where the severity of cases varies widely, resulting in potential selection bias compared with the general population. On the maternal side, the cohort included many women who experienced massive hemorrhage or required emergency surgery. A small number of participants had psychiatric symptoms, most of whom showed EPDS ≥9 on day 3; however, they were included in the analysis to reflect real-world perinatal conditions. On the neonatal side, the cohort also included preterm infants, congenital anomalies, and cases requiring admission to the NICU. Therefore, compared with the general population, our cohort may have contained a higher proportion of women with elevated EPDS scores.

## Conclusions

This study demonstrated that EPDS scores on postpartum day 3 are significantly correlated with those at one month. Early screening may facilitate the identification of at-risk mothers and allow earlier intervention. Furthermore, even when the EPDS score is negative on day 3, scores of ≥5 warrant careful monitoring given the risk of later conversion to positive.
